# Modified Intracellular-Associated Phenotypes in a Recombinant *Salmonella* Typhi Expressing *S*. Typhimurium SPI-3 Sequences

**DOI:** 10.1371/journal.pone.0009394

**Published:** 2010-02-24

**Authors:** Patricio Retamal, Mario Castillo-Ruiz, Nicolás A. Villagra, Juan Morgado, Guido C. Mora

**Affiliations:** 1 Departamento de Medicina Preventiva Animal, Universidad de Chile, Santiago, Chile; 2 Departamento de Ciencias Biológicas, Universidad Andres Bello, Santiago, Chile; University of Massachusetts Medical School, United States of America

## Abstract

A bioinformatics comparison of *Salmonella* Pathogenicity Island 3 sequences from *S*. Typhi and *S*. Typhimurium serovars showed that ten genes are highly conserved. However three of them are pseudogenes in *S*. Typhi. Our aim was to understand what functions are lost in *S*. Typhi due to pseudogenes by constructing a *S*. Typhi genetic hybrid carrying the SPI-3 region of *S*. Typhimurium instead of its own SPI-3. We observed that under stressful conditions the hybrid strain showed a clear impairment in resistance to hydrogen peroxide and decreased survival within U937 culture monocytes. We hypothesized that the *marT-fidL* operon, encoded in SPI-3, was responsible for the new phenotypes because *marT* is a pseudogen in *S*. Typhi and has a demonstrated role as a transcriptional regulator in *S*. Typhimurium. Therefore we cloned and transferred the *S*. Typhimurium *marT-fidL* operon into *S*. Typhi and confirmed that invasion of monocytes was dramatically decreased. Finally, our findings suggest that the genomic and functional differences between SPI-3 sequences have implications in the host specificity of Typhi and Typhimurium serovars.

## Introduction

The *Salmonella enterica* genome has five DNA regions that are related to pathogenicity and are shared by all serovars, referred to as the *Salmonella* pathogenicity islands (SPI). The acquisition of these SPI regions seems to have played a major role in the adaptation of *Salmonella* to new niches in its vertebrate hosts, supporting many bacterial requirements during infection [1]. One such island, SPI-3, is located in the *selC* locus of *S. enterica* and contains ten ORFs [2], some of them have been experimentally associated with virulence functions in this pathogen. This is the case of the *mgtCB* operon, for which there is evidence of involvement in intramacrophage survival and virulence in mice [3]–[5]. This operon is encoded in all *Salmonella* serovars within a very conserved SPI-3 region [6]. The *mgtC* seems to be a pathogenic factor that has been repeatedly acquired by horizontal gene transfer throughout bacterial evolution, because it has also been related to virulence in *Mycobacterium tuberculosis* and *Brucella suis* [7]–[9].

Other SPI-3 genes that have a role in pathogenicity are *misL* and *marT.* The *misL* gene encodes an autotransporter protein that is involved in the adhesion of *S. enterica* serovar Typhimurium (*S*. Typhimurium) to the extracellular matrix in mice and chicks, thereby acting as an intestinal colonization factor [10], [11]. It has also been shown that *marT* encodes a transcriptional regulator that activates the expression of *misL* [12]. However, both MisL and MarT are absent from the virulence repertoire of some *Salmonella* serovars, because their nucleotide sequences have acquired translational stop codons and are considered pseudogenes. This is the case in *S. enterica* serovar Typhi (*S*. Typhi), a human-specific pathogen that causes Typhoid fever, a life-threatening and systemic infection of higher incidence in developing countries [13].

The remaining SPI-3 ORFs encode conserved hypothetical proteins that have not been experimentally characterized [14].

There are three pseudogenes in the published SPI-3 sequence of *S*. Typhi CT18 [15], which is consistent with previous reports establishing that almost 5% of the *S.* Typhi gene repertoire is composed of pseudogenes, many of which are encoded in pathogenicity islands [4]. The disruption or inactivation of this amount of genome sequences suggests that *S.* Typhi has lost the ability to express several virulence-associated functions, a condition of biological relevance that may explain the invasive nature and human-restricted host specificity of this pathogen.

To further contribute to the growing knowledge of the SPI-3 in *Salmonella* and the functional effects of the structural differences observed, we transferred the complete *S*. Typhimurium SPI-3 island into a *S.* Typhi clinical strain and then characterized some of the phenotypic effects on the pathogenicity of the recombinant strain. The main findings were related to a higher susceptibility to oxidative stress and a lower survival rate inside human monocytic cell line. In order to elucidate some of the protein functions involved in these phenotypes, we cloned the *S*. Typhimurium *marT-fidL* operon in a low copy number plasmid. When present in a *S*. Typhi wild-type strain, this operon significantly affected the early survival of bacteria inside monocytic cells, giving a partial explanation for the phenotypes described with the recombinant SPI-3 bacterium.

## Materials and Methods

### Bacterial Strains and Culture Media

A clinical isolate of *S.* Typhi, designated STH2370, was obtained from the Infectious Diseases Hospital Lucio Córdova in Santiago, Chile. The strain has been described previously [16], and was isolated directly from the blood sample taken from a typhoid patient, grown to overnight density, and frozen as a primary stock. The *S.* Typhimurium reference strain 14028s was obtained from the Instituto de Salud Pública, Santiago, Chile.

The strains were grown routinely in liquid culture in Luria-Bertani (LB) medium (Bacto Tryptone, 10 g/L; Bacto Yeast Extract, 5 g/L; NaCl, 5 g/L) adjusted to pH 7 (NaHPO_4_/NaH_2_PO_4_ 25 mM), at 37°C, with aeration provided by shaking, or anaerobically, prior to invasion assays in cultured human cells [17]. When required, the medium was supplemented with kanamycin (Kan; 50 µg/mL), chloramphenicol (Cam; 20 µg/mL), and/or gentamycin (Gem; 10 µg/mL). Media were solidified by the addition of agar (15 g/L).

### Construction of a *S*. Typhi Genetic Hybrid Containing the SPI-3 Region of *S*. Typhimurium

**Figure 1:**
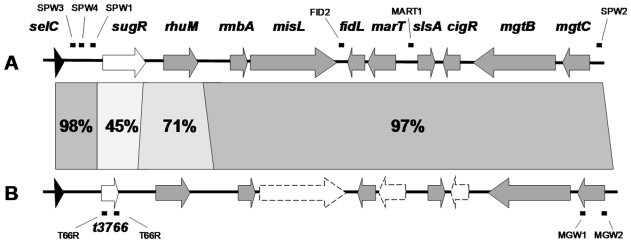
Genomic organization of Salmonella pathogenicity island 3. A, SPI-3 in *S*. Typhimurium. B, SPI-3 in *S*. Typhi. ORFs are depicted by arrows and tRNAs appear as closed triangles. ORFs with no orthologs between these serovars and pseudogenes are depicted by white and segmented arrows respectively. The percentage (%) of nucleotide identity of the SPI3 loci is shown in between. The aligning sites of the PCR primers are shown by black dots.

To characterize the new properties conferred on the *S.* Typhi STH2370 strain upon the introduction of the entire *S.* Typhimurium SPI-3 region, the exchange of this complete island sequence was performed by homologous recombination using both the lambda Red recombinase system [18] and a generalized transduction with the P22 HT105/1 *int201* phage [19]. Briefly, the Kan^R^ cassette was amplified with the primers SPW3 (5′-AGAGAAAGTGGAATATTATTTTATTAATCACCATTTACCGTGTAGGCTGGAGCTGCTTCG-3′) and SPW4 (5′-CGTTAGTCCTGGCGTACTTTTATCCCTTCTTTGCAGACGACATATGAATATCCTCCTTAG-3′), and was inserted at the left end of the *S.* Typhimurium 14028s SPI-3 island (Fig. 1). The Cam^R^ cassette (chloramphenicol resistance, codified in the pKD3 plasmid), amplified with primers MGW1 (5′-ATGGAGGAACGTATGTTAATGTTTCCTTATATTTTAAATTTGTAGGCTGGAGCTGCTTCG-3′) and MGW2 (5′-TGACCCACGAGCTCGGCACGAATTTCTTTATAGCCCTGTTCATATGAATATCCTCCTTAG-3′) was placed at the right end of the *S*. Typhi STH2370 SPI-3 island (Fig. 1). A P22 HT105/1 *int201* phage lysate of the 14028 Kan^R^ strain was subsequently produced and used for the generalized transduction of the STH2370 Cam^R^ strain. *S.* Typhi clones that had acquired the Kan^R^ but lost the Cam^R^ phenotype were then selected. Using this procedure the hybrid *S.* Typhi SPI-3 STm (SPI-3 STm) strain was obtained, and the exchange of the pathogenicity island sequences was confirmed by PCR (data not shown).

To exclude the possibility that the *S*. Typhimurium SPI-3 flanking sequences might be contributing to the phenotypic modifications in the *S*. Typhi transformants, the 14028s ΔSPI-3::*aph* mutant strain was constructed using the lambda Red recombinase system [18] with the primer pair SPW1 (5′-AACGCAGGCGCTACGTTTGTCGATGCCGTAACTTTCTGAATGTAGGCTGGAGCTGCTTCG-3′) and SPW2 (5′- GCTAAATATAGCACGTACTTATTCTTCCAGAAAAAATGGACATATGAATATCCTCCTTAG-3′) (Fig. 1). Later the P22 HT105/1 *int201* phage lysate obtained from this strain was used to transduce STH2370 cells. The transference of this mutation was also confirmed by PCR.

### Construction of Mutant Strains

The mutant strains were constructed using the lambda Red recombinase methodology, as previously described [18]. The primer pairs used were T66W1 (5′- TTCTTGCTGGAAAAACTGGCGTGTAATGCTGAAGAATTCATGTAGGCTGGAGCTGCTTCG-3′) and T66W2 (5′- TTTATTCATTGCCACCTCCCTGACTGTTCTCGTTATTTTGCATATGAATATCCTCCTTAG -3′) for the S. Typhi *t3766* mutant strain, CIGW1 (5′- CAAATCGTTTTCTAACGAGGTGACGCCATGCAAAAAAGAGCATATGAATATCCTCCTTA -3′) and CIGW2 (5′- ACGATTTTCCACTCATAGCCCGGATAATAAGGTAATTGTCTGTAGGCTGGAGCTGCTTCG -3′) for the SPI-3 STm *cigR* mutant strain, MISW1 (5′- ATGACCTTGTTATCAAACCAGTTGTACAGCATCCAGGTATCATATGAATATCCTCCTTA -3′) and MISW2 (5′- TCGGCCAATATTTACGTCGGCGACGATCTGTATATCAAAATGTAGGCTGGAGCTGCTTCG -3′) for the SPI-3 STm *misL* mutant strain, and the primers MARW1 (5′- TTTTATAAGGTTGCTGACAAATCAATGCCGTAACCGCTGTGTAGGCTGGAGCTGCTTCG -3′) and MARW2 (5′- CTACGTTATGTTTTGGCATGGTAACAATGCGATGTTATGGCATATGAATATCCTCCTTA -3′) for the SPI-3 STm *marT* mutant strain.

### Phenotypic Analyses

For the assay of growth in minimal medium, bacteria were cultured overnight in M9 medium [20] supplemented with 10 mM MgCl_2_, 0.2% glucose, tryptophan and cysteine (50 µg/mL each). Then the culture was washed three times with Mg^+2^ free medium, diluted 1/200 in M9 media containing 10 µM or 10 mM MgCl_2_, and incubated with shaking at 37°C. For the assay of growth in acidic pH, bacteria were grown in LB broth pH 7 at 37°C overnight with shaking, then were washed three times with LB at pH 5 (citric acid 0.1 M), diluted 1/200 in the same medium and incubated with shaking at 37°C. In both assays the bacterial growth was measured with a spectrophotometer at an optical density of 600 nm (OD_600_) for several periods.

To determine bacterial survival after exposure to oxidative stress, the strain was cultured in 2 mL of LB pH 7 at 37°C overnight with shaking. Then the culture was diluted 1/100 in LB pH 7, the H_2_O_2_ added to a final concentration of 3 mM and the culture incubated at 37°C for 30 min with shaking. For the survival rate analysis, aliquots of cultures were diluted and plated in triplicate before and after challenge. Survival was expressed as the percentage of colony forming units (CFU) after the assay compared to the count before the challenge.

### Infection Assays in U937 Human Monocytic Cells

Infection of U937 cells was carried out as described previously [5]. Briefly, cells were grown in Dulbecco's modified Eagle's medium supplemented with 10% (vol/vol) fetal bovine serum, seeded into 24-well tissue culture plates at a concentration of 10^5^ cells per well, and then incubated at 37°C in 5% CO_2_ until confluent growth was achieved. Later the cells were centrifuged and washed three times with PBS. Approximately 2×10^6^ to 5×10^6^ CFU of exponential-phase (OD_600_, 0.15 to 0.20) anaerobically grown bacteria were pelleted, washed twice with PBS, and resuspended in 1 mL of PBS. Aliquots (100 µL) of bacteria were added to U937 cells at a multiplicity of infection (MOI) of 50:1. After 1 h of infection, cells were centrifuged and washed three times with PBS, and the medium was replaced with Dulbecco's modified Eagle's medium supplemented with 10% (vol/vol) fetal bovine serum containing Gem (200 µg/mL). After additional incubation for 1 h U937 cells were washed three times with PBS and lysed with 0.5% deoxycholate for the early survival evaluation (time 2 h) or were incubated for 23 h in fresh medium containing Gem (25 µg/mL) for late survival evaluation (time 24 h). When assayed Gem resistant strains, the antibiotic used was Kan (50 µg/mL for 1 h and 20 µg/mL thereafter) instead of Gem. The titers of intracellular bacteria were determined by serial dilution of cell lysates on agar plates. The percentage of survival was calculated at 2 h considering the initial inoculate as 100%, and at 24 h considering the CFU counted at 2 h as 100%.

### Cloning and Complementation of the *marT-fidL* Operon

To clone the *marT-fidL* operon, we amplified a 2.1-kb fragment of serovar Typhimurium 14028s DNA that included the operon coding sequence and 699 bp of the *marT* upstream region using the primer pair MART1 (5′-CTGCAGCTGATGACAGTAATCCGTTG-3′) and FID2 (5′-GAATTCTGATCTTCAGCGGCTTTTAC-3′) (Fig. 1). The PCR fragment was purified, digested with endonucleases PstI and EcoRI, and ligated to the same sites of the Gem^R^, low copy-number plasmid vector pBBR-5 [21]. The ligation mixture was electroporated into *E. coli* DH5α, and the white colonies that grew on LB medium plates containing Gem were screened. Plasmid DNA purified from one clone yielded an insert of the predicted size after digestion with the same PstI and EcoRI endonucleases (data not shown), and was later electroporated into *S*. Typhi strain STH2370.

### RNA Isolation and RT-PCR

Total RNA was extracted and purified using Trizol and was treated with RNase-free DNase I (amplification grade; Gibco-BRL). RT-PCR was performed with 500 ng of DNase-treated RNA using the Superscript reverse transcriptase (Invitrogen). Amplification was performed for 30 cycles (94°C for 40 s, 55°C for 40 s, and 72°C for 1.5 min, followed by a 10 min extension at 72°C). The primers used were RTMART1 (5′- AAACCGTCTCGATAACCGCAT-3′) and RTMART2 (5′-TCCAGAAATGAATCGCCTGG-3′) corresponding to an internal region of the *marT-fidL* sequence, and the universal primers 8F and 1498R were used to amplify 16S rRNA [22]. The transcript of the *S*. Typhi *t3766* gene was amplified with the primer pair T66D (5′-TCAAAATCTCGCAGCCAGCG-3′) and T66R (5′-AAATTAATGCAGGGGCGTGG-3′) (Fig. 1). Genomic DNA was used as a positive control, and DNase-treated RNA that had not been reverse transcribed was used as a negative control. The PCR product was electrophoresed on 1% agarose gels with ethidium bromide.

### Statistical Analyses

Statistical analyses were performed using the Student's t-test for independent samples. Values of P<0.05 were considered significant. This test was performed using Microsoft Excel® software.

## Results

### The *S.* Typhi Genetic Hybrid Containing the SPI-3 Region of *S.* Typhimurium Has Modified Pathogenicity Associated Phenotypes

Following alignment, comparison of the published SPI-3 homolog sequences from *S*. Typhi and *S*. Typhimurium showed a high degree of nucleotide identity (92% on average), although this was not homogeneously distributed (Fig. 1). The central and right regions from both islands appear conserved between these serovars and the most remarkable differences are a highly polymorphic region at the left end of the island and the existence of pseudogenes in the *S*. Typhi sequence, as mentioned previously.

**Figure 2:**
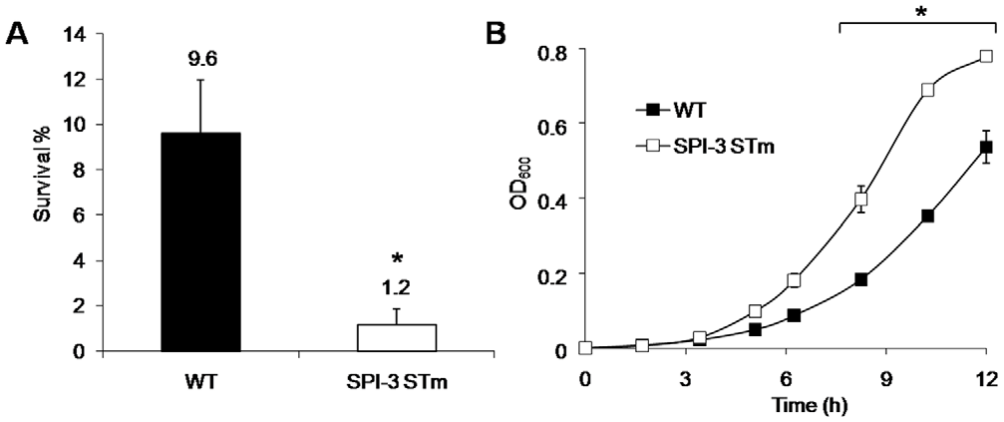
Phenotypic assays with a *S*. Typhi genetic hybrid strain. A, Percentage (%) of survival following 30 min exposure to hydrogen peroxide (3 mM). B, Growth at pH 5, based on the optical density at 600 nm (OD600) at different times (h). The strains used were a *S*. Typhi STH2370 containing the SPI-3 region of *S*. Typhimurium (SPI-3 STm) and the STH2370 wild-type strain (WT).Values represent the mean of at least three independent experiments ± SD (*p<0.05).

**Figure 3:**
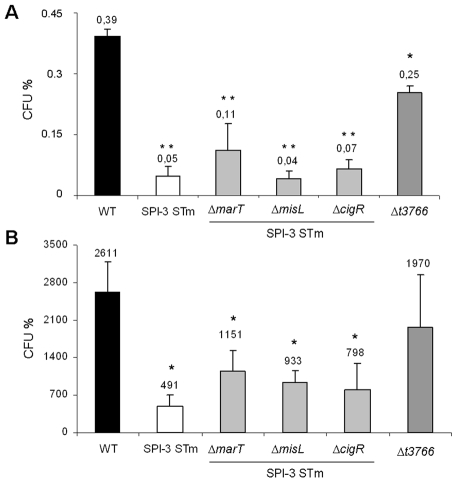
Infection assays in human cells. A, early survival (2 h). B, late survival (24 h). Percentage of colony forming units (CFU%) inside U937 human cells. The strains used were the STH2370 wild-type strain (WT), a *S*. Typhi STH2370 expressing the SPI-3 of *S*. Typhimurium (SPI-3 STm), the SPI-3 STm strain containing deletions of *marT* (Δ*marT*), *misL* (Δ*misL*) and *cigR* (Δ*cigR*) sequences, and a *S*. Typhi STH2370 *t3766* mutant strain (Δ*t3766*). The values represent the mean of at least three independent experiments ± SD. Different numbers of asterisks represent significant differences between results (p<0.05).

To determine the pathogenicity functions that are associated with the polymorphic sequences of SPI-3, we constructed a *S*. Typhi hybrid containing the SPI-3 region of *S*. Typhimurium instead of its own sequence (SPI-3 STm strain). To validate these findings, we chose three recombinant clones and the parental STH2370 wild-type strain and compared their survival or growth behavior under stressful conditions, including survival to oxygen and nitric oxide derived radicals, to starvation and to hyperosmolarity and growth in acid and basic media, at different temperatures, in anaerobiosis and growth in a low Mg^2+^ environment Surprisingly, there were three significantly altered phenotypes exhibited by the SPI-3 STm strain: an 8-fold lower survival rate after 30 min of exposure to 3 mM hydrogen peroxide (Fig. 2A), a 1.5-fold greater growth rate after 12 h of incubation in pH 5 LB broth (Fig. 2B), an 8-fold and 5-fold lower survival rate inside U937 monocytic cell line (2 h and 24 h, respectively), compared to the wild-type *S*. Typhi strain (Fig. 3).

All recombinant clones consistently had the same growth and survival rates in the assays, demonstrating that the experimental strategy for the SPI-3 exchange was a robust procedure for determining the phenotypes reported here.

In the control STH2370 ΔSPI-3::*aph* strain, a unique finding was the inability to grow at a low magnesium concentration (10 µM Mg^2+^) (data not shown). This result was expected because the *mgtCB* operon was among the deleted genes in this clone, and is responsible for adaptation to low-Mg^2+^ environments in both *S.* Typhimurium [3] and *S.* Typhi [5]. Hence, the modified phenotypes described in the SPI-3 STm hybrid strain were caused by genes contained in the *S.* Typhimurium SPI-3 island when expressed in the context of the *S.* Typhi genome.

To study the involvement of the pseudogenes in the modified phenotypes of SPI-3 STm strain, we constructed three strains deleting one of the following genes *misL*, *marT* and *cigR.* However, these mutations did not show any difference in the survival to peroxide and the growth rate in pH 5 (data not shown) nor in the survival rate inside U937 cells (Fig. 3) in comparison with the hybrid strain. This result suggests that other sequences, alone or in combination, are modifying the behavior of the *S*. Typhi SPI-3 STm strain.

### The *S.* Typhi *t3766* Mutant Strain Has a Lower Early Survival Rate inside Human Cells

Because the *t3766* sequence is not codified in the *S.* Typhimurium SPI-3 island, it is absent from the SPI-3 STm hybrid strain. To determine whether this deletion could have some effect in the studied phenotypes, we constructed a *S*. Typhi *t3766* mutant strain that was tested under the same experimental conditions. A lower survival rate inside U937 cells at 2 h post infection was the unique significant finding (p<0.05) that had this *t3766* mutant in comparison with the *S*. Typhi wild type strain. However, this susceptibility was not similar to the observed with the SPI-3 STm strain (Fig. 3A), suggesting that additional factors are contributing to the lower intracellular survival of the hybrid strain.

### Characterization of the *S*. Typhimurium *marT-fidL* Operon

**Figure 4:**
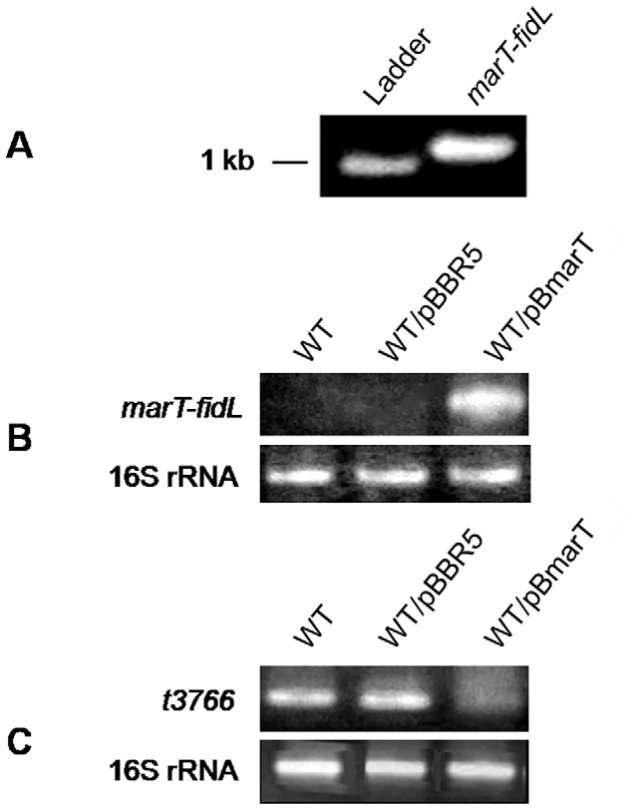
Reverse-transcription PCR assays. A, Amplification of the *marT-fidL* internal sequence with RNA extracted from *S*. Typhimurium. B, Amplification of the *marT-fidL* internal sequence. C, Amplification of the *t3766* sequence. In B and C the RNA was extracted from strains STH2370 wild-type (WT), STH2370/pBBR-5 (WT/pBBR5) and STH2370/pBmarT (WT/pBmarT). All strains were grown up to the stationary phase, and the 16S rRNA amplification was used as the control.

An *in silico* analysis of sequences from *S*. Typhimurium SPI-3 suggested that the main factor responsible for the phenotypic changes observed in the SPI-3 STm hybrid could be *marT*, since its protein product exhibits similarity to the putative DNA binding domain of the CadC transcriptional activator of *E. coli* K-12, which has been associated with the acid stress response [2], [12], [23]. In our current study, we sequenced the *marT* gene from the clinical *S.* Typhi strain STH2370 and confirmed that the same number and positions of the internal stop codons were present as described in published *S.* Typhi Ty2 and CT18 genomes [24]. In addition, we confirmed that *marT* and *fidL* are co-transcribed in *S*. Typhimurium strain 14028s (Fig. 4A), this is the reason for cloning the operon and its promoter in the pBBR-5 plasmid. When *S*. Typhi was transformed, the transcription of *marT-fidL* in the STH2370/pBmarT (*marT-fidL* ^+^) strain was confirmed by RT-PCR (Fig. 4B). To determine whether the operon could modify the expression of a gene codified in the SPI-3 of the STH2370/pBmarT strain, we aligned the reported binding site of MarT in the *misL* promoter [12] against the *S*. Typhi SPI-3 sequence. The highest identity (55%) was found in the upstream region of *t3766*. In addition, there are two ToxR consensus binding sites (TTTTGAT) localized both within the *t3766* promoter region and inside its coding sequence. Given that MarT is a member of the family of ToxR-like regulatory proteins [12], *t3766* is a candidate target for regulation by the *marT-fidL* products. The RT-PCR assay showed that the *S*. Typhimurium *marT-fidL* operon represses the transcription of this *S*. Typhi SPI-3 sequence (Fig. 4C).

### The *S.* Typhimurium *marT*-*fidL* Operon Modifies the Early Survival of *S.* Typhi in U937 Monocytic Cell Line

**Figure 5:**
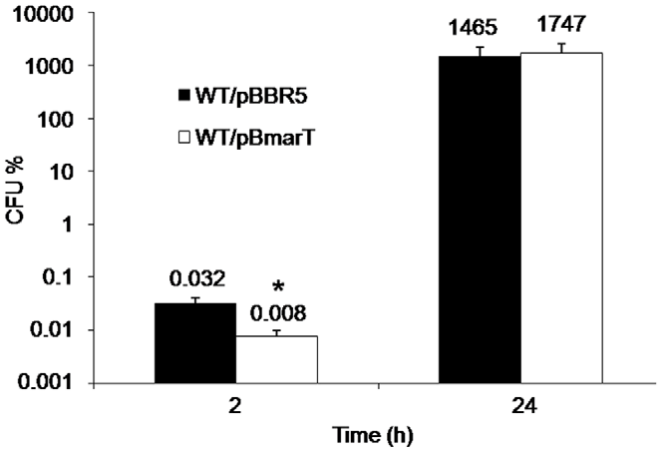
The *S*. Typhimurium *marT-fidL* operon modifies the invasiveness of *S*. Typhi to U937 human cells. Percentage of colony forming units (CFU%) after invasion (2 h) and proliferation (24 h) inside U937 human cells. The STH2370 wild-type strain was transformed with either the pBBR-5 (WT/pBBR5) or pBmarT (WT/pBmarT) plasmids. Values represent the mean of at least three independent experiments ± SD (*p<0.05).

To determine whether the *marT-fidL* products can affect the phenotypes described in the SPI-3 STm strain, the STH2370/pBmarT strain was evaluated in the same assays. The only significant phenotype arising as a result was a four-fold lower survival rate after two hours of infection in monocytic U937 cells, showing the same intracellular survival as the wild-type when evaluated at 24 h post infection (Fig. 5).

## Discussion

The ability to infect and live within an animal host suggests the existence of multiple pathogenic functions codified in the bacterial genome, destined to increase fitness under a stressful environment or to subvert the host response.

Pathogens with a broad host range obviously require more mechanisms to evade the immune response than those with a single host. The variety of such additional mechanisms will be in accordance with the multiplicity of bactericidal mechanisms displayed by the immune system of susceptible individuals.

In the case of *Salmonella* serovars, which have a highly similar genetic background, the differences in host range suggest a variable number of pathogenic functions codified on the chromosome, although the size of the chromosomes among serovars is very similar. This genetic trait is clear when comparing the serovars Typhi and Typhimurium, with the *S*. Typhi pseudogenes being the main cause of this characteristic [4]. However, this global loss of functions observed in *S*. Typhi probably implies better adaptation to life within the human body, as it is not exclusively localized in the intestinal tract, as is the case with *S*. Typhimurium [25].

It is possible that SPI-3 and its described variability among *Salmonella* serovars [6] could participate in their respective host specificity and invasive nature by differentially affecting the bacterial response to some stressful conditions. Here we suggest two such responses that could be codified on the SPI-3: adaptation to a pH 5 and survival following hydrogen peroxide damage. Hence, it is probable that the island has a important role in the intracellular life of *Salmonella* by acting not only in response to magnesium scarcity [2], [3], [5], [26], but also to reactive oxygen species and acidic environments. However, not all SPI-3-associated responses will be useful and functional in all serovars because when some of them are expressed in certain strains, they could have a negative effect on pathogenic associated phenotypes. This could be the case of a *S*. Typhi strain expressing the SPI-3 of *S*. Typhimurium. Given the gain of function that should follow the exchange of genes in place of pseudogenes, our first hypothesis was that the recombinant strain could be more resistant to some stressful conditions. However, only growth in acidic medium supported this hypothesis because in the other two assays (survival in response to 3 mM H_2_O_2_, and infection of U937 monocytic cell line) the recombinant strain appeared significantly more susceptible than the wild-type STH2370 strain. These findings strongly suggest that pseudogenes were preserved to increase *S*. Typhi pathogenicity in the human host.

The specific sequences participating in the phenotypes exhibited by the recombinant SPI-3 STm strain were not determined, although the products of the *marT-fidL* operon can be implicated by modifying the early survival during infection of U937 monocytes (Fig. 5). This effect could be partially explained by the ability to represses the transcription of *t3766* (Fig. 4C), a sequence codifying a product with an unknown function that participates in the early survival of *S*. Typhi within monocytic cells (Fig. 3A). However, the presence of the *marT-fidL* operon confers a higher susceptibility to intracellular survival than that observed with the *t3766* mutant strain, suggesting that other mechanisms are involved and demonstrating the regulatory role of the operon with a potential ability to modulate bacterial responses.

MarT is a member of the family of ToxR-like regulatory proteins, which are involved in the activation of genes repressed by the histone-like nucleoid structuring (H-NS) protein [12], and silences genes with GC contents lower than the genomic average by restricting the access of RNA polymerases to the DNA [27]. Some previous reports and the results of present study suggest that a functional and structural homology exists between MarT and ToxR as i) MarT can bind to the *misL* promoter region, which has a low GC content (34.8%) [12] and is also recognized by H-NS proteins [27]; ii) ToxR is a transcriptional regulator that is constituted by periplasmic transmembrane and cytosolic domains [28]. *In silico* analyses of MarT suggest that this protein has the same domains described for ToxR (data not shown). iii) ToxR can sense external stimuli and bind DNA to directly modify the expression of the regulated genes. In *Vibrio cholerae*, *toxR* is co-transcribed with *toxS*, a regulatory product that acts as a chaperone-like protein and stabilizes the periplasmic domain of ToxR, thus modifying on its function [28], [29]. In the case of *marT*, it is co-transcribed with *fidL*, and the phenotypes observed in *S.* Typhi require the presence of both genes. When only *marT* was cloned and transfer into *S*. Typhi, none of the phenotypes were observed (data not shown); and iv) ToxR recognizes the TTTTGAT sequence in the promoter regions of target genes [30]. Interestingly, there are some repeats of this consensus binding site in the SPI-3 coding region of both *S.* Typhi and *S.* Typhimurium. In this work it was found that the *S.* Typhimurium *marT-fidL* operon can repress transcription of *S*. Typhi *t3766* (Fig. 4C), an SPI-3 ORF that contains the consensus TTTTGAT in both promoter region and the coding sequence.

Some AT-rich pathogenicity islands encode H-NS antagonists [31], [32], indicating that these genetic elements have developed counter-mechanisms to evade H-NS-mediated silencing [27]. Although other experiments are required to elucidate the role of the MarT and FidL proteins, it is tempting to speculate that they belong to the same group of transcriptional regulators that are encoded in mobile genetic elements and act on AT-rich sequences to modulate their expression in receptor bacteria. In this hypothetical situation, the expression of MarT in *S*. Typhi could have unexpected effects on several pathogenic associated phenotypes, probably decreasing the virulence of this human restricted serovar.

In this work the expression of *S*. Typhimurium SPI-3 sequences in *S*. Typhi indicated previously unknown functions of the island by clearly modifying some phenotypes of the recombinant strains. This suggests that pathogenicity islands are evolving in each serovar of *Salmonella* with particular characteristics, affecting their virulence and host specificity. It is interesting that there are conserved and variable regions within the SPI-3 coding sequence among serovars [6], probably determining functions that are similarly required in different host species and other functions that respond to bacterial requirements in specific hosts.

Finally, the results of this work demonstrate that the transference of sequences with unknown function between serovars is an experimental strategy that could help in finding keys with which to address the study of these poorly known genetic islands.
